# Coating Materials to Increase the Stability of Liposomes

**DOI:** 10.3390/polym15030782

**Published:** 2023-02-03

**Authors:** Diana Pasarin, Andra-Ionela Ghizdareanu, Cristina Emanuela Enascuta, Catalin Bogdan Matei, Catalin Bilbie, Luciana Paraschiv-Palada, Petronela-Andreea Veres

**Affiliations:** 1 Institutul National de Cercetare-Dezvoltare pentru Chimie si Petrochimie, ICECHIM, 202 Splaiul Independentei, 060021 Bucuresti, Romania; diana.pasarin@gmail.com (D.P.); cata.matei@gmail.com (C.B.M.); 2 Facultatea de Stiinta si Ingineria Materialelor, Universitatea Politehnica din Bucuresti, 313 Splaiul Independentei, 060042 Bucharest, Romania; 3 Expergo Business Network SRL, 6 Radu Calomfirescu, 030216 Bucuresti, Romania; catalin@expergo.ro (C.B.); luciana@expergo.ro (L.P.-P.); petronela@expergo.ro (P.-A.V.)

**Keywords:** coating, drug, food, liposomes, stability

## Abstract

Liposomes carry various compounds with applications in pharmaceutical, food, and cosmetic fields, and the administration route is especially parenteral, oral, or transdermal. Liposomes are used to preserve and release the internal components, thus maintaining the properties of the compounds, the stability and shelf life of the encapsulated products, and their functional benefits. The main problem in obtaining liposomes at the industrial level is their low stability due to fragile phospholipid membranes. To increase the stability of liposomes, phospholipid bilayers have been modified or different coating materials have been developed and studied, both for liposomes with applications in the pharmaceutical field and liposomes in the food field. In the cosmetic field, liposomes need no additional coating because the liposomal formulation is intended to have a fast penetration into the skin. The aim of this review is to provide current knowledge regarding physical and chemical factors that influence stability, coating materials for liposomes with applications in the pharmaceutical and food fields to increase the stability of liposomes containing various sensitive compounds, and absorption of the liposomes and commercial liposomal products obtained through various technologies available on the market.

## 1. Introduction

Liposomes can be defined as artificial spherical nanostructures that consist of one or more phospholipid bilayer membranes [1,2,3].

They are the most commercialized active substance delivery systems with a prime encapsulation capacity and therapeutic index, biocompatibility, biodegradability, flexibility, and safety due to their structural similarity to cell membranes (lipid bilayer) [4]. The liposome structures consisting of phospholipids come from various sources of lecithin (soy, egg yolk, sunflower, rice beans, and rape seed). The main phospholipids used are phosphatidylcholines, phosphatidylethanolamines, phosphatidylserines, and phosphatidylglycerols. These lipids are amphiphilic because they have a group of polar heads and a lipophilic tail, making them suitable for use in obtaining liposomes [5,6].

The aqueous core of liposomes is enclosed by the phospholipid bilayer. The core encapsulates the water-soluble drugs, and the hydrophobic domain entraps insoluble compounds. Due to their structure, their stability, bioactivity, and bioavailability in the human body are increased [7,8]. Liposomal nanostructures can have one or more layer membranes.

The number of layers influences the liposome size from 20 nm to 1000 nm [9]. Based on size and lamellarity, liposomes are classified into several categories: small unilamellar vesicles (20–100 nm) [10], large unilamellar vesicles (>100 nm) [11,12] giant unilamellar vesicles (>1000 nm) [13,14], oligolamellar vesicles (100–1000 nm) [15,16], multilamellar large vesicles (>500 nm) [17,18] and multivesicular vesicles (>1000 nm) [19].

The methods used to obtain liposomes can be grouped into conventional and modern methods. The conventional methods used are thin-film hydration, ethanol/ether injection, reverse phase evaporation (detergent depletion, microfluidic channel, heating, membrane extrusion, high shear homogenization, and sonication), and the proliposome method. The modern methods used are freeze drying, dual asymmetric centrifugation, and supercritical fluid methods (supercritical anti-solvent, supercritical CO_2_ reverse phase evaporation process, the rapid expansion of a supercritical solution, depressurization of an expanded liquid organic solution suspension, and super-critical assisted liposome formation) [20].

It is recommended that the encapsulation of the active ingredients in liposomes be conducted by appropriate methods to protect them both from the food processing conditions and the action of the gastrointestinal tract (GT) juices to ensure targeted, controlled release. Different categories of active substances can be encapsulated in liposomes, with applications in the pharmaceutical, cosmetic, and food industries, such as vitamins, enzymes, antimicrobial polypeptides, essential oils, phenolic compounds, minerals, antioxidants, food additives, flavors, fatty acids, drugs, toxins, proteins (peptides), antigens, and nucleotides [21,22,23,24].

The use of the liposome-based delivery system in the pharmaceutical field is predominant because the therapeutic effect is achieved by reducing the dose and frequency of administration of the encapsulated compounds [25]. Currently, there are various liposomal products on the market with applications in the pharmaceutical and nutraceutical fields, such as Abhope, Liposomal Vegan D3 K2 Magnesium, VENTUS liposomal Omega 3, LVC5, and others In cosmetics, there are many commercial nano-liposomal products.

Liposomes and niosomes are particularly interesting due to the phospholipid component that has a high-esterified essential fatty acid content. Phosphatidylcholine is preferred as an essential component in obtaining liposome membranes because of its conditioning and softening properties. Unilamellar liposomes are most often used in the cosmetic field, and they do not need an additional coating because they must have an easy and fast penetration into the skin. Studies have shown that liposomes are suitable for delivering active compounds due to their compatibility with the skin and their extended and slow dermal release.

Firm Christian Dior SA (Paris, France) launched into the commercial market Capture anti-aging cream, and Laboratories RoC (Istanbul, Turkey) launched Myosphere—the first emulsion with the inclusion of liposomes and the first liposomal facial cream for men [26].

In food supplement formulations, liposomes can provide protection and the controlled release of sensitive active substances.

The encapsulation of various active compounds leads to the enrichment and strengthening of various food supplement formulations [27]. In the food industry, liposomes are used in various applications, such as enhancing the bioavailability of nutritional components, providing antimicrobial activity of the encapsulated ingredients, increasing intestinal absorption, improving flavors, and extending shelf life. There are very few food commercial applications of liposomes. They have been applied in different food matrices, namely dairy, meat, beverages, chocolate, and candy [28,29].

Liposomes are ideal delivery systems, but their use is limited by their short circulation half-life and their susceptibility to oxidation and hydrolysis. Their physical stability can be affected by gastric juice’s low pH, bile salts that solvate the liposomes, and enzymatic hydrolysis resulting in their breakage and release of the loaded molecules in the GT. There are many methods to increase the stability of liposomes, such as the application of a protective coating, changing the liposomal membrane structure, the addition of cryoprotectants before liposome lyophilization, and the use of surfactants or various special polymer gels [9,30,31,32]. Common cryoprotectants used to improve the stability of liposomes during lyophilization include dimethyl sulfoxide, glycerol, sugars and disaccharides, and polyampholytes. These materials help protect the integrity of liposomes during low-temperature storage by preventing the formation of harmful ice crystals [33].

To improve the stability of liposomes against the hostile environment of the GT, it is effective to modify their surface by the layer-by-layer electrostatic deposition method.

A variety of polymers, such as chitosan, pectin, alginate, etc., have been applied to coat the surface of liposomes with an impact on their functionality, namely (1) extending the circulation time, which improves the transport and release of active substances; (2) the reduction in oxygen exposure and implicitly the decrease in lipid oxidation due to the thick polymer layer; and (3) the decrease in the leakage of loaded components [34].

This review aims to provide current knowledge regarding coating materials for liposomes with applications in the pharmaceutical and food fields and their influence on the stability and absorption of liposomes.

## 2. Physical and Chemical Factors Influencing Liposome Stability after Being Obtained

The main problem with the application of liposomes at the industrial level is their low physical and chemical stability due to fragile phospholipid membranes and their peroxidation. Physical degradation can sometimes be due to changes in the structure of the liposomes.

The stability of the liposome structure influences the controlled release of active compounds, both in the bloodstream and in the intestinal mucosa, depending on the route of liposome administration (injectable or oral). Liposome stability is influenced by physical factors (storage temperature and light) and chemical factors (lipid peroxidation and variations in pH).

### 2.1. Physical Factors

Storage temperature is an important factor that influences the stability of liposomes. Studies have shown that liposomes with different compounds lose their stability at high temperatures. The range of experimental temperatures varied between −150 °C and 50 °C, and the testing time was between 24 h and 90 days [35,36]. Thermal liposomal stability depends on both the encapsulated active compound and the liposomal membrane structure. The thermal instability of peptide-loaded liposomes can limit their incorporation in thermally processed food [37].

The photostability of liposomes has been tested by exposing them to various sources of light (UV-A, UV-B, and UV-C) or radiation. The most common source used was sunlight exposure under different conditions for a testing period from 4 h to 6 months [38,39].

Details regarding the influence of physical factors on the stability of the liposomes are given in Table 1.

As shown in Table 1, the liposomes were degraded by almost 36% by irradiation with various UV rays (UV-A, UV-B, UV-C), and the stability of liposomes under direct natural sunlight decreased by 25%.

The photostability of liposomes is reduced or degraded due to the breakdown of lipids caused by exposure to direct sunlight, and fluorescent or ultraviolet lights that can cause the oxidation of lipids and other compounds. Photostability can alter the structure of liposomes by affecting the interaction between their hydrophobic and hydrophilic regions. This alteration can influence the permeability, stability, and other properties of the liposome.

### 2.2. Chemical Factors

The chemical stability of liposomes can be influenced by the oxidation process of the phospholipid components (lipid peroxidation), which can occur as a result of the obtaining process (purification), sterilization, or even storage conditions [49]. Lipid peroxidation refers to the oxidative degeneration of lipids, characterized by forming free radicals in the lipid tails, such as cyclic peroxides and hydroperoxides [50]. The free radicals from fatty acids function as intrinsic compounds, unsaturated fatty acids being more susceptible than saturated fatty acids to this process [51].

During the obtaining process, oxidation can occur in different stages, depending on the method used. The methods that involve the use of organic solvents (ethanol, methanol, chloroform, ether, and methylene chloride) facilitate the process of molecular dispersion of lipids, and encapsulation is thus conducted with high efficiency. The destabilization and initiation of the oxidation process are influenced by the temperature at which residual organic solvents are removed in the final stage [52,53].

Since the most common route of administration of various compounds encapsulated in liposomes is oral, they must be sterilized by various processes including steam heating (autoclaving), ultraviolet and gamma ionizing irradiation, chemicals, and filtration methods. All of these methods can lead to the oxidation of the liposomal membrane [54]. For example, steam heating results in phase structural transitions (degradation and/or leakage of the encapsulated compounds) as well as oxidation of the phospholipidic component due to the high temperatures at which it is achieved (T > 121 °C) [55]. Gamma ionizing irradiation uses a high energy ionizing power and has a strong penetration capacity. This type of sterilization can destabilize the liposomal membrane in several ways: peroxidation of lipids (unsaturated lipids), hydrolysis or lipid fragmentation components, and pH changes in the solution [56].

The stability of liposomes under storage conditions, regarding pH variations, is crucial for the delivery of pH-sensitive compounds. Liposomes are usually obtained in a neutral buffer solution so that the active compounds are also in a neutral medium after incorporation into the liposomes [57]. Experiments were performed in which the liposomes were stored under different conditions of pH 2.5–10.5 for 30 min to 30 days to determine the optimal storage pH range to maintain long-term stability. The studies regarding the influence of pH on liposome stability are given in Table 2.

As shown in Table 2, the stability of liposomes decreases by 50% when the pH of the solution is acidic and by 20% when the pH increases above neutral conditions. The pH of the liposomal solution can affect the stability, size, and permeability of the liposomes as well as the release rate of the substances embedded in the liposomes.

The researchers have shown that the stability of liposomes is maintained if certain conditions of obtaining and storage are respected. The liposomal solution pH must be neutral; the production and sterilization methods used have to avoid the use of organic solvents and lipid peroxidation processes. The liposomes obtained must be kept refrigerated, at a neutral pH, and protected from direct sunlight.

The liposome membranes can be protected from the process of lipid peroxidation or this type of reaction can be minimized when liposomes are kept under inert gases such as nitrogen or argon (there is practically minimal exposure to oxygen) or if the liposomes are lyophilized before storage. Companies such as LipoCellTech™ (Soest, The Netherlands) and LivOn labs (United States of America) maintain the storage stability of their liposomal commercial products by the lyophilization process or by maintaining the liposomes in a semiliquid form with the addition in the composition of thickeners and/or emulsifier agents (xanthan gum and Tween ™ 80).

Peroxidation can also be minimized by storing liposome formulations in light-resistant containers or by removing heavy metals that may be present as a result of the obtaining process [60]. In addition, the addition of cholesterol to obtain the liposomes leads to rigidifying of the phospholipid bilayers and thus inhibits lipid peroxidation induced by the addition of copper in the reaction medium [61].

## 3. Coating Materials for Liposomes with Applications in Drug Delivery

Liposomes used as nanocarriers with amphiphilic character can deliver different drugs by their encapsulation. In the bloodstream, they are treated as foreign objects and are destroyed by immune system cells by phagocytosis [62]. Protecting liposomes from the rapid reaction of the immune system and increasing their stability against low pHs in the stomach represent challenges.

To improve and achieve a good biological interaction (with blood and tissues) while using liposomes as carrier drugs, studies have been conducted to improve liposomes’ surface. Liposomes were coated with film-forming compounds to improve the stability of their membranes.

The most common materials used for coating and the change in the surface of liposomes are found in the following classes of compounds: saccharides and their derivatives, polymers, and proteins. The general coating materials for liposomes encapsulating drugs and their properties are illustrated in Table 3.

Saccharides and their derivatives tend to be more physically and chemically stable, resulting in site-vesicle structures specific to their biological environments. In the coating process of liposomes using saccharides, the greatest influence on the permeability, fluidity, and integrity of the membrane is given by the mechanisms (adsorption, coagulation, and bridging) and by the method of binding the saccharide to the lipid bilayer of the surface [123].

Following the use of various saccharides (chitosan, alginate, pectin, starch, and others) and their derivatives as coatings, researchers observed improvements in the structural and physical-chemical properties of liposomes as well as an increase in the biochemical stability related to biological stimuli, such as pH, osmotic pressure, ionic strength, and temperature. A prolonged release of active substances has been observed with increased bioavailability and biostability for saccharide-coated liposomes compared to uncoated liposomes [57,68,73].

Chitosan is one of the most exploited polymers in biomedical science for drug delivery, gene delivery, and tissue engineering. Its main advantages are its mucoadhesive capacity, stability of labile drugs in the GT, bioavailability, and the controlled release of the drug [124].

The use of chitosan as a coating material has also several limitations; as it increases the size of the vesicles and the viscosity in the liposomal solution, it requires a low pH for its solubility (it is insoluble at the physiological pH of 7.4), and it is also difficult to remove excess chitosan from the liposome surface [125,126,127,128,129,130].

Physical-chemical changes in the structure of chitosan (i.e., molecular weight, crosslinking with anions, dextran, sulfates, tripolyphosphate, or covalent binding of functional groups) can contribute to overcoming limitations and extend its use in many applications. Interest in the structural changes in chitosan has led to the development, in recent years, of groups of derivatives with improved chemical, biological, and functional properties. The solubility of chitosan was increased by the introduction of alkyl (hydroxypropyl or carboxymethyl) groups into its structure. The trimethylation of the primary amino groups of chitosan improved its mucoadhesive properties and reduced trans-epithelial electrical resistance, while thiolation enhanced the gelling properties and permeability [124,131].

Among the recent applications of chitosan derivatives, we mention a few. Thiolated chitosan liposomes loaded with insulin, administered orally, have been tested under in vitro and in vivo conditions, with promising results in intestinal epithelial mucosal biodistribution and bioavailability [132]. Trimethyl chitosan nanoparticles in combination with fucoidan (hypoglycemic) inhibited α-glucosidase activity [133]. Chitosan crosslinking with sodium tripolyphosphate liposomes containing carvedilol exhibited a better bio-accessibility and antihypertensive effect [134].

The main challenges in the future development of chitosan-based liposomes (chitosomes) are safety and site-specific drug targeting.

Very rigid xanthan gum promotes the static stability of liposomes by resisting the Brownian motion of the lipid droplets, as an additional benefit, while the flexible guar gum prevents contact between the liposome vesicles. Their synergistic effects have been shown to improve the stability of the liposomal system during long-term storage. The use of gums as coating materials has the disadvantage of obtaining a viscous system, requiring more attention to the concentration of polysaccharides because, at high gum concentrations, the gel network could be damaged and, consequently, there may be an agglomeration of liposome vesicles and leakage of the bioactive substance [51,135]. Xanthan gum, as an anionic dietary fiber, can inhibit lipid oxidation by transferring its ability to bind iron ions (the process occurs faster at a pH of 3.5 than at a pH of 7, which may be due to the increased solubility of iron under acidic conditions), but a significant increase in the rate of lipid aggregation was observed under gastrointestinal conditions [136].

Alginate-coated liposomes have improved stability in the gastric environment, but in some cases, the interactions between them significantly alter the permeability of the membrane; calcium ions seem to induce a higher drug leakage than sodium ions [137].

Polymers are widely used as a coating material because they are more stable than phospholipids, and their properties can be imprinted on liposomes by controlled synthesis. The advantage of using polymers is their intercalation in the lipid secondary layer due to their varied block structure and because they reduce the predisposition of lipids to oxidative degradation [138]. Polymers are preferred as liposome coatings because of their improved mucoadhesive properties that can prolong the retention and enhance the adhesion and penetration of drugs through the gastrointestinal barriers [139]. For example, liposomes coated with charged polymers, such as poly-l-lysine or poly-l-arginine, are electrostatically repellent and stable in colloidal systems; the coating also serves as a barrier that controls the release rate of active compounds. However, a strong polymer–liposome interaction can cause leakage of the encapsulated active compounds, while a weak attachment can cause the polymer to detach after injection, leading to a shorter circulation time [140].

Recent research pursuing the modification and coating of liposome membranes by using film-forming polymers (PEG-polyethylene glycol, Eudragit EPO, poly (L-lysine), and others) has led to increased stability in the organism, showing properties of precise targeting and delayed release of drugs. PEG has been the most widely used coating material for liposomes carrying antitumor drugs due to its hydrophilic properties, molecular flexibility, and neutrality, which can prolong circulation time and reduce reticuloendothelial system (RES) clearance [141].

However, recent studies have shown that PEGylated products may cause an immune response (both intravenous and oral), such as hypersensitivity, cytoplasmic vacuolation, accelerated blood clearance, and antibody production under certain conditions. Recently, several potential safety issues have been raised from the repeated administration of these types of liposomal products because the use of high molecular weight PEG, which human enzymes cannot effectively degrade, leads to its accumulation in the body.

The use of lower molecular weight PEG has various toxic side effects. Liposomal drug formulations and their storage stability may be influenced by PEG’s instability under conditions of exogenous stress, such as heat, radiation, or mechanical forces [142,143,144,145].

A new kind of PEG derivative, which has two ends grafted with cholesterol (cholesterol–PEG–cholesterol, CPC), was reported by Wang, et al. [146]. This derivative reduces the uptake by phagocyte cells and extends the circulation time. Sadzuka, et al. [147] showed that a reduction in nanoparticle opsonization can be achieved by combining PEG with different-sized chains that modify the conformation of the polymer on the surface of the liposomes.

Many polymers have been proposed to stabilize liposomes as an alternative to PEGylation: polyglycerol-modified liposomes, the use of super-hydrophilic zwitterionic polymers such as poly (carboxy betaine), and a triblock non-ionic surfactant (Pluronic F127), largely used as a food and drug additive [148,149,150].

Lane, et al. [151] hypothesized that glycosaminoglycan (GAG) heparosan (HEP; [-4-GlcA-β1-4-GlcNAc-α1-] n), a natural polysaccharide, may serve as a PEG alternative for coating liposomes. The coated drug-carrying liposomes with HEP are protected from the mononuclear phagocyte system, extending liposome circulation time and potentially avoiding immune-mediated clearance.

Liposomes coated with natural macromolecules (natural biodegradable proteins), which are recognized by the body, can change the shield surface and the layers of the liposome membrane, having the same binding way, being a good alternative for the replacement of PEG coating materials [152].

Proteins (albumin, gelatin, silk fibroin (SF), and others) used as liposome coatings and drug-binding molecules improved the long blood retention of the active compounds and sustained permeation along with a high capacity of controlled targeting [96,100,101].

By combining groups of coating materials (chitosan–pectin, pectin–whey protein, PEG–chitosan, and others) the physical-chemical properties such as solubility, thermal protection, resistance to oxidative stress, and adequate cell protection have been improved [106,107,111,116].

Liposomes coated with dimethyl amino methyl-dextran, silica, and ceramics showed better skin permeation and oxygen protection along with increased photovoltaic stability and anti-interference capacity [85,119,120,122].

Challenges in using coating materials to improve liposome stability in drug delivery include controlling the uniformity of the coating, optimizing the surface area-to-volume ratio, and ensuring that the coating is compatible with the lipid components of the liposomes. This can be complicated due to the variability and complexity of natural lipids. In addition, certain coating materials may cause toxicity or other undesirable effects, which is a potential problem when they are used in pharmaceutical applications. Therefore, experimental methods are required to ensure that the coating materials provide the desired stability.

## 4. Coating Materials for Liposomes with Applications in the Food Industry

In the food industry, the application of liposomes mainly focuses on texture alteration and water retention improvement. In recent years, studies have been conducted on the encapsulation of food components using various technologies (LipoCellTech, Kerkplein, The Netherlands, stealth liposomes, or non-PEGylated liposome technology). Active ingredients must be formulated in such a way as to protect them against production technology and environmental conditions so that they can be safely delivered to the targeted organs and cells. The results of various studies have shown that the coating of liposomes with different types of compounds by creating a layer on the surface of the membrane and providing electrostatic repulsion has led to increased physical stability, resistance to mechanical stress, and a low release speed of charged compounds [153].

Different types of materials have been used in the coating of liposome carriers for food-active compounds. Two groups of compounds have been used most frequently due to their food-grade qualities: saccharides and their derivatives and proteins. These groups of compounds have been chosen for their biocompatibility, low or nontoxicity, and neutral organoleptic properties [154].

The general coating materials for liposomes with applications in the food industry and their properties are illustrated in Table 4.

Saccharides and their derivatives studied as coatings for food ingredients entrapped in liposomes have been shown to improve the thermal, physical and chemical, functional, and structural stability of liposomes during storage, with better release during in vitro digestion [82,155,156].

Some researchers [108] synthesized vitamin C and introduced it in mandarin juice. The multi-layered liposomes were the result of depositing positive chitosan and negative sodium alginate on the surface of the anionic nanoliposomes. The coated structure of nanoliposomes modified the surface characteristics of these. After 90 days, the vitamin C was still protected, and no significant organoleptic changes were observed in the fortified samples.

Low methoxyl pectin (LMP) can be used as a macromolecular material to modify the surface of liposomes. The liposomes formed not only have a good particle size and potential, but also have better stability during long-term storage. The double layer is protected from oxidation and maintains a good active compound release efficiency. LMP-coated liposomes added to orange juice create a bridge with metal ions and form a network-like gel to establish the stability of the liposomes. The addition of pectin with a different degree of esterification leads to an increase in the particle size of the liposomes. This is mainly due to the adsorption of pectin on the surface of the liposomes. Some environmental factors, such as pH, ionic strength, and temperature, have a significant effect on the appearance, particle size, and flow rate of liposomes, but LMP as a coating material can have a protective effect [161,162].

Inulin-coated liposomes showed better stability in the presence of surfactants and electrolytes. The use of cationic inulin helps to create a physical barrier to prevent the aggregation and fusion/coalescence phenomena, while using long-chain inulin has been shown to increase liposome stability during storage and improve gastric viability [163]. Coating liposomes with lactose or inulin prevents their rapid dissolution in alcohol, providing a protective effect. In addition, because lactose is a molecule present on the surface of blood cells, the affinity avoids macrophages. Depending on the microstructural order and the molecular weight of the saccharide, the structure of the liposome changes; for example, inulin as a coating material confers longer release properties due to its higher molecular weight. Thus, the thickness of the liposomes is closely related to the molecular weight; the higher the molecular weight, the thicker and more resistant the liposomes, which last longer in the gastrointestinal tract [154,163]. The use of proteins as a coating material has led to the enhancement of thermal and light stability with an improvement in the active compound stability under gastric conditions. Whey protein isolates used as a coating material for astaxanthin entrapped in liposomes improved the bio-accessibility and protected the liposome membranes against alteration during in vitro digestion [93,94]. The use of chitosan in combination with other compounds (alginate) to obtain a liposome coating material led to an improvement in the antimicrobial and prolonged antioxidant activity of the encapsulated compounds and the improvement of thermal and light storage stability [108,113,158,159].

Challenges in using coating materials to improve the stability of liposomes when used in the food industry include the need to ensure that the coating material is safe for consumption, the cost and complexity of manufacturing, the consistency of liposomal properties during storage, and the difficulty in determining the optimal parameters for coating. In addition, some modifications to the liposome formulation may be required to ensure that the liposomes remain stable over a long period of time and can survive during storage, handling, and shipment.

## 5. The Influence of Polymer Coatings on the Absorption of Liposomes

The stages of digestion for liposomes as vehicles for drug or bio-compound delivery have been studied under in vitro conditions in simple mono-compartment to complex multi-compartment dynamic digestive systems. In addition, an artificial gastric digestive system with a 3D-printed shape was developed and validated to follow the food digestion mechanisms [164].

The oral administration of liposomes raises several challenges, namely susceptibility to physiological factors in the GT, poor permeability of liposomes across gastrointestinal epithelia (the main absorption barrier), and liposomal formulations (manufacturing). Under the action of physiological factors, liposomes composed of phospholipids and cholesterol lose their integrity (are unstable), and the active ingredients are released but not in the target cell or tissues [165].

The liposome delivery systems cross the GT and change until they reach the intestinal mucosa where absorption takes place. Only some of the ingested liposomes reach full form and are absorbed by the lymph pathway [4].

At the level of the stomach, acid-stable gastric lipase-initiated digestion takes place. This can slightly affect the structure of the liposomes because they have a lipid bilayer membrane and because cholesterol from the structure increases the rigidity of membranes. So, they decrease slightly in diameter due to the pressure difference between the inside and outside of the two sides of the liposomes [34,166,167]. When the simulated digestion time was extended to 120 min, aggregation of the liposomes was observed due to the reduction in the electrostatic repulsion force between the liposomes under the conditions of a low pH. The encapsulated compounds, such as betacyanins, lutein, and β-carotene, could be degraded slower or faster without affecting the liposomes by crossing the liposomal membrane and exposure to gastric acid [168].

Most of the liposome digestion takes place in the small intestine under the action of pancreatic enzymes (colipase-dependent pancreatic lipase acts on unhydrolyzed triacylglycerols from the stomach, pancreatic lipase-related protein 2 acts as phospholipase and galactolipase, carboxyl ester hydrolase, bile salt-stimulated lipase, hydrolyses cholesterol esters, triacylglycerols, monoacylglycerols, vitamin (A, E) esters, phospholipids carotenoid esters, galactolipids, and polyethylene glycol mono- and di-esters, and pancreatic phospholipase A2, involved in the digestion of phospholipids, catalyzes the hydrolysis of the sn-2 fatty acyl ester bond of 3-sn-glycerophospholipids) [169].

Bile salts mediate digestion in several ways: (i) they weaken the interfacial stresses between molecules by facilitating the action of phospholipase A2 and lipase on the liposomal lipid phase; (ii) they weaken the structure of the phospholipid bilayers by the insertion of bile salt molecules and the formation of channels, which make the membranes more susceptible to the lipolysis process by fluidizing them; (iii) bile salts facilitate the hydrolysis of phospholipids and the release of fatty acids by the adsorption of lipase to phospholipid bilayers; (iv) they increase lipolysis by eliminating the accumulation of fatty acids and increasing the accessibility of lipase; and (v) they aid in the solubilization and absorption of the lipolysis products by forming mixed micelles [170,171].

When biopolymers are deposited on the surface of liposomes, their properties in different GT fluids may change the ability of enzymes to act on the surface of the lipids, which could improve the digestive stability of the liposomes. Their digestion in the GT involves a complex set of physical-chemical and biochemical reactions that affect the uptake of hydrophilic and hydrophobic-loaded molecules [172].

The role of chitosan in liposome digestion and absorption is still controversial. Some authors [172,173] report that the polycationic nature of chitosan prolongs the retention time through the intestinal mucosa. The explanation is that mucin, an anionic glycosylated protein negatively charged at the mouth pH, covers the chitosan-coated liposomes, offering further protection to the loaded active molecules during the other digestion phases. It takes place in the electrostatic interaction between the amine group (NH3+) of chitosan and the carboxylate (COO^−^) or sulfonate (SO3^−^) group of mucins. In addition, the adhesion of chitosan-coated liposomes to the mucosal membranes, negatively charged, enhances; so, the bioactive compounds are more available for absorption and the half-time of clearance increases.

The mechanism responsible for the permeation is based on the positive charges of this polysaccharide, which structurally reorganizes (opens) the tight junctions of the mucosal cell membrane proteins, facilitating the paracellular transport of hydrophilic macromolecules. Chitosan’s molecular weight and degree of deacetylation influence the increase in membrane permeability. Thus, a high degree of deacetylation and a high molecular mass contribute to the increase in the chitosan charge density, which leads to the increase in epithelial permeability and implicitly to the increase in drug transportation [174].

Highly methoxylated pectin is a widely used liposomal coating because it increases the stability of liposomes during storage and adheres to the intestinal epithelium without influencing membrane permeability [4].

Coating liposomes with PEG increases their intravenous circulation time and increases the stability of liposomes at the intestinal level through a mechanism of adhesion to the mucus of intestinal epithelia. The adhesion mechanism of positively charged mucoadhesive polymers is based on the ionic interaction between them and negative compounds from the mucus layer [175,176].

The polymer coating is a promising way to modify the surface characteristics of the vesicle’s stability to improve its applicability [177].

The liposome content is delivered in the cell by four mechanisms [178]. The first mechanism is the adsorption of liposomes on cells which can be specific—through specific receptors on the cell membrane and liposomes—and nonspecific, realized through attractive forces. The second mechanism represents the exchange of lipids between the cell membrane and the liposomal membrane due to their similarity. The third mechanism is endocytosis (for large particles by phagocytosis and receptor-dependent internalization by pinocytosis). The fourth mechanism is the fusion between the plasma and liposomal membranes. The liposomal content is delivered directly into the cell [179,180]. The liposome membrane is broken, and the encapsulated active compounds are released; these can be internalized into the cell in three ways: simple diffusion, facilitated diffusion, and active transport [181].

The cell uptake of liposomal oral or injectable products can be influenced by the liposomes’ size and surface charge. Experiments with liposomal formulations with surface charge and varied lipid compositions have shown that anionic or neutral liposomes are efficiently absorbed by monocyte-derived DCs [168,182]. Depending on the size of the liposomes, they follow different pathways. Studies show that liposomes between 40.6 nm and 276.6 nm in diameter are up-taken by Caco-2 cells [183].

From a pharmacokinetic perspective, the main goals of liposome drug delivery systems are improved in vivo drug release profiles, including enhanced drug absorption, targeted drug delivery, a modified metabolic pattern, a prolonged residence time of the drug in the body (e.g., in the bloodstream), and delayed and/or reduced renal excretion of the drug. From the initial stages of liposome system design through to the final clinical evaluations, absorption, distribution, metabolism, and excretion (ADME) must be considered to accurately understand the pharmacokinetic properties of this drug delivery system. In terms of ADME affecting the pharmacokinetic behavior of the drugs, for liposome delivery systems, the lipid bilayers serve as barriers between aqueous compartments and distribution compartments [184].

A quantitative explanation of the in vivo conditions under which a drug dose leads to therapeutic or side effects is provided by pharmacokinetics. For this purpose, the drug concentrations in the biophase and/or toxic phase must be considered. The concentration–time curves of drugs serve as the basis for pharmacokinetic research, which in turn serves as the starting point for estimating pharmacokinetic parameters with the help of corresponding mathematical models [185].

These factors should provide a quantitative link between biological concentrations and drug effects. In this situation, liposomes as drug carriers can be used as “pharmacokinetic modifiers” to achieve predetermined spatial and/or temporal targeted drug delivery. Recently, coated liposomes have been developed for the targeted delivery of therapeutic drugs to increase oral drug bioavailability, solubilize drugs for intravascular delivery, maintain the effects of drugs or genes in target tissues, reduce the potential for toxic effects or adverse reactions, and/or improve the stability of therapeutic drugs against enzymatic hydrolysis or other particular nutrients, peptides, and nucleic acids [186].

Liposomes, due to their subcellular size, can penetrate the tissue through the capillary walls and cross epithelial tissues and are usually taken up by cells. A therapeutic concentration must be achieved in the target tissues by modulating the physicochemical properties of the liposomes, as unfavorable exposure of nontarget tissues to these drugs can potentially lead to adverse effects.

Various characterization experiments are often performed during the development of these nanocarriers, mainly in vitro and in vivo tests, to optimize the drug delivery of liposome systems. Particle size, shape, chemical composition, surface hydrophilicity, polarity, drug release profile, and other physicochemical characteristics are used in in vitro studies to provide an indirect measurement of the drug delivery capabilities of different compounds [187].

On the other hand, successful in vitro tests are followed by in vivo studies to test the liposome drug carriers for efficacy in a living, intact organism or in specific organs or tissues. The produced drug nanocarriers are often subjected to two different types of in vivo experiments: pharmacodynamic tests on the pharmacological effects and pharmacokinetic studies for the expected effects of the particle and/or the drug associated with the particle.

Coated and uncoated liposome drug carriers often consist of a large number of individual parts that interact as integrated systems in a special structure. Compared to the free drug, these different components, especially the therapeutic portion (drug), have different ADME properties (absorption, distribution, metabolism, and excretion). Therefore, the localization of the drug and coated liposome drug carriers in the biological system is a problem in ADME-related animal studies with coated liposome drug carriers. In vivo studies in which each component is independently tracked may not be sufficient to determine the therapeutic efficacy and toxicity of coated liposome carriers [188,189].

Therefore, instead of studying only one component, different pharmacokinetic parameters of both components, i.e., the drug and the carrier, should be used. Several coated liposome formulations are being investigated in various stages of clinical trials for medical applications; recently, several coated liposome formulations have been used in ongoing clinical trials. These clinical trials and their applications are listed in Table 5.

As the clinical trials conducted with coated liposomes are at different stages, it can be stated that each study mainly investigates the occurrence of adverse effects after the administration of liposomal formulations. According to the ADME literature studies, events such as low oral bioavailability, unfavorable clearance of nanoparticles by RES, excretion of the drug via urine in parenteral administration, and entrapment and elimination of circulating carriers by opsonization are the typical obstacles encountered in many routes of administration and the first processes analyzed.

Coating liposomes with certain materials can alter the ADME of the drug they are designed to deliver. Advantages of coating liposomes include increased protection of the drug from degradation, improved permeation through cell membranes, and the ability to control the release of the drug at a specific rate. The main disadvantages of coating liposomes are the impairment of pharmacokinetics, bioavailability, and the biological half-life of the drug, the difficulty in determining the optimal parameters for coating, and the possible toxic effects of the coating material. Toxicity depends on the material used, but general potential toxic effects include inflammation and irritation of skin and tissues, an increase in antigenicity, an increased risk of sensitization and allergic reactions, and disruption of cell membrane barrier functions.

## 6. Patent Applications and Patents on Coated Liposomes

There are recent patent applications related to coated liposomes, but not all of them have been granted as patents. In addition, most of these patents relate to pharmaceuticals and not to the food industry, as different legal requirements apply to patenting a drug delivery system. Patents on food delivery systems are less common and often require additional evidence of the efficacy of such systems before they can be granted. Some of the recent patent applications and patents involving coated liposomes are listed in Table 6.

It is anticipated that patents regarding coated liposomes with applications in the food industry will be published in the future due to the lack of current representation in this sector.

## 7. Liposome Commercial Products Available on the Market

The purpose of encapsulating active compounds is to maximize their bioavailability and health benefits through controlled release. Therefore, there is a growing demand for smart delivery systems that allow controlled delivery at the right time and place; the only essential requirement is that this delivery system is produced depending on the route of administration.

The liposome products available on the market showed greater stability, biological efficacy, and health benefits due to their synergistic properties [190]. A short selection of commercial products containing different active compounds encapsulated in liposomes are illustrated in Table 7.

The encapsulation technologies used in the development of liposomal pharmaceuticals are stealth liposome technology, DepoFoam™ technology, heat-sensitive liposomes, and non-PEGylated liposome technologies. Only in stealth technology (PEGylation), the structure of the liposome membrane is modified or coated, and the polymer used is polyethylene glycol (PEG). This technology is the most common for the development of cancer therapy drugs (such as Doxil and OnivydeTM) because it makes it difficult for mononuclear phagocytes to detect the liposomes [191,192,193,194].

Liposomal food supplements or food products are obtained by technologies that are designed to protect the liposomes from heat, high pressure, or chemicals. Hypernatura and LivOn labs use LIPOCELLTECH™ or cold-process liposomes for the food liposomal supplements obtained. The liposomes obtained by these technologies do not use a coating material [195,196].

Currently, there are only two food products on the market, in the form of functional teas, which contain active ingredients encapsulated in liposomes, obtained by the technology described in the patent of the company Bio-Up Mimetic Technologies Inc. The liposomes have no coating, but the method of obtaining them has provided optimal stability and high efficiency from an economic point of view [197].

## 8. Conclusions

Liposomes, due to their biocompatibility and encapsulation capacity, are a promising delivery system for different compounds. The integrity of phospholipid membranes ensures the stability of liposomes. The rate and degree of peroxidation and hydrolysis of phospholipids are major factors that determine the shelf life and performance of liposomes for medical or food applications. The hydrolysis of phospholipids can especially affect cholesterol-free liposomes used, in particular, in thermosensitive liposome formulations [198]. Physical and chemical factors decrease the integrity of lipid bilayers, and the liposomes are destabilized.

The most used coating material for liposomes with pharma applications is PEG, but considering the accumulation effect in the body—due to the frequent use of liposomes coated with PEG—new materials are sought.

In the food field, saccharides, their derivatives, and proteins are frequently studied as liposomal coating materials for their nontoxicity and neutral characteristics.

Stability is important for liposomes because, depending on the degree of stability, the compounds transported by liposomes reach the site where absorption takes place. If stability is low, then a large part of the compound is degraded along the route, and if stability is very high, then the compound cannot be released. This makes stability crucial in achieving the purpose for which the compound has been encapsulated in liposomes.

However, most of the recent studies on liposomal surface modification, particularly for phospholipid modification, are related to the pharmaceutical, medical, and cosmetic industries. Research on the in vitro digestion stability or interaction mechanism of liposomes with surface modifications in food technology is very rare.

## 9. Outlook

In recent years, different types of liposomes coated with various materials have been developed to increase the stability, protection, and controlled/targeted release of active compounds. These liposomes can be used in pharmaceutical, food, and cosmetic applications to increase the bioavailability of nutrients. Better solutions are still needed to improve the quality and shelf life of liposomes containing functional compounds.

A big challenge for liposome production at the industrial level is to maintain their optimal stability both in terms of physical factors (maintaining optimal conditions for manufacturing and storage), but also in terms of chemical factors (oxidation reactions that may occur during the obtaining process). However, it must also be taken into account that the technological process of obtaining liposomes needs to be economically efficient with a low number of nontoxic chemical reagents used.

Another aspect to consider is that liposomes, regardless of the route of administration, have to be protected from the immune system’s rapid reaction and gain increased stability against the low pHs in the stomach for the targeted delivery of active compounds.

The number of pharma-liposomal products on the market is higher compared to liposomal food supplements and nutraceuticals. As for food products that contain liposomes, they are at the laboratory research level with a very small number of products on the market. The liposomal beverages on the market are obtained without thermal processing and are kept in refrigerated conditions (optimal conditions for maintaining the stability of the liposomes). An additional challenge is to diversify the range of liposome-containing foods, which are obtained through a production technology that involves thermal processes, high pressure, or additional sterilization, such as bars, breakfast cereals, and instant noodles.

In the future, it is targeted to achieve the development at the industrial level of solid food products containing liposomes and their commercialization as well as the increase in liposome stability through the development of new coating materials.

## Figures and Tables

**Table 1:** Influence of physical factors on liposome stability.

Factors Range/Value	Stability Measurements	Testing Time	Active Compounds Encapsulated in Liposomes	Bibliography
**Temperature**				
4 °C, 25 °C, 37 °C	75.54%, 67.57%, 20.28% retention rates	21 days	Betacyanin	[40]
20–47 °C	3.47–4.33% release	20 days	Piperine	[41]
−150 °C, −80 °C, −25 °C (Liquid nitrogen)	76–90%, 75–88%, 37% retention	90 days	Carboxyfluorescein	[35]
4 °C, 25 °C	14.25%, 40.04% loss in nanoliposomes	21 days	Red cabbage anthocyanins	[42]
Room temperature	30–90% degradation of extract	21 days	Black carrot extract	[43]
4 °C, 25 °C	96.81%, 8.82% encapsulation efficiency	90 days	Phenylethyl resorcinol	[44]
4 °C, 50 °C	89.43% retention rate	30 days	Curcumin	[45]
4 °C, 25 °C	90%, 80% encapsulation efficiency	30 days	Afatinib	[46]
4 °C	95% change rate of vesicle size	21 days	Curcumin	[47]
**Light**				
UV-A (12.86 W/m^2^), UV-B (15 W/m^2^), UV-C (14.29 W/m^2^) irradiation	Instability to UV irradiation: UV-A < UV-B < UV-C; 25.841%, 32.881%, 35.678% degradation for small unilamellar vesicles liposomes	4 h	Fulfonamides, sulfamethoxypyridazine, sulfachloropyridazine	[38]
UV light irradiance (0.35 W/m^2^)	88% retention	6 h	Curcumin	[47]
Natural sunlight	75.21% retention rate	180 days	Quercetin	[39]
Direct sunlight; sun UV irradiation through window glass; outdoors under the shade	Completely degraded	24 days	Silver sulfadiazine	[48]
UVA irradiation	100–36.5% degradation	48 h	Phenylethyl resorcinol	[44]
Fluorescent lamp (20 Watt) or dark	49.44% improved storage stability	30 days	Curcumin	[45]
D65/ID65 emission Standard (artificial daylight fluorescent lamp, indoor indirect daylight)	Not available	30 days	Anti-inflammatory drugs	[36]

**Table 2:** Influence of pH on liposome stability.

pH Value	Stability Measurements	Testing Time	Active Compounds Encapsulated in Liposomes	Bibliography
3, 4.3, 5, 7	50%, 80%, 80%, 80 retention rates	21 days	Betacyanin	[48]
5.5, 6.0, 6.4, 7.4	Not available	30 min	Doxorubicin hydrochloride	[58]
5.5, 7.4	Cationic liposomes 63.6%, nontargeting liposomes 28.1%, pH-sensitive liposomes 35.6%—release rate in neutral pH 7.4	4 h for acidic conditions, 24 h for alkaline conditions	Afatinib	[46]
2.5, 5.0, 7.4	55%, 43%, 34% encapsulation efficiency post-incubation	6 h	Curcumin	[59]
4.5, 7.4, 10.5	Completely degraded at day 18 acidic, neutral conditions, degraded at day 30 alkaline conditions	30 days	Silver sulfadiazine	[48]
5, 6, 7, 8, 9	>80% acidic conditions retention rate, <50% neutral and alkaline conditions retention rate	30 days	Curcumin	[45]

**Table 3:** Coating materials for liposomes as drug delivery means.

Coating Material	Type of Encapsulated Drugs	The Advantage of the Coating Material Used	Bibliography
**Saccharides and their derivatives**			
Chitosan	Immunoglobulin/mupirocin/Curcumin/sumatriptan/mucoadhesive loratadine/pelargonidin-3 -O-glucoside	- Increased bioactivity, bioavailability, and biostability during the oral digestion of the drug - Increased the capability of delaying the release of the drug in gastrointestinal simulated digestion after obtaining a high encapsulation efficiency in the liposome for the lowest particle size - Improved the physical properties of the liposomes	[63,64,65,66,67,68]
Poly-galacturonic acid	Nisin	- Controlled release of antimicrobial peptides - Increased the encapsulation efficiency and thermal stability of the liposomes	[69,70,71]
Sodium alginate	Calcitonin/Perilla Oil	- Increased the resistance of the membrane structure, the bioavailability, and the prolonged release of the drug - Provided good chemical stability and in vitro release behavior of the active compound	[72,73]
Calcium alginate	Oxaliplatin/Acid Folic/Ampicillin/Metformin	- Prolonged the release of the drug and enhanced the control of liposome dimension distribution - Increased entrapment efficiency and enhanced stability of liposomes	[74,75]
Pectin	Resveratrol/Amoxicillin/Tagitinine C/Phlorizin	- Improved the long-term release of drugs and system stability - Presented small-sized liposomes with a slightly slower release - Improved immobilization, encapsulation efficiency, as well as physical storage stability of liposomes	[57,76,77]
Starch	Fasudil	- Increased the storage stability and in vitro release of the active compound - Enhanced the uptake of liposomes and had a high entrapment efficiency	[78]
Guar gum	Hydrophobic bioactive compounds/Vitamin D3	- Improved the liposome membrane stability (reduced membrane degradation after simulated digestion) - Increased thermal and light stability of the liposomes with a prolonged storage stability	[79,80]
Xanthan gum	Dioctadecyl dimethylammonium bromide	- Improved the release profile of the low-stability drug	[81]
Cationic inulin	Betaine/carvone	- Improved the thermal, physical, and oxidative stability of liposomes; provided in vitro sustained release of the active compound	[82]
Galactomannan	Ascorbic acid	- Presented good encapsulation efficiency and improved the stability of the drug - Sustained the release of the active compound under gastrointestinal pH conditions	[83]
Hyaluronic acid	Antitumoral drug delivery/anti-melanoma agents (dacarbazine and eugenol)	- Reduced immune response and improved the pharmacokinetics of the drug - Presented a good stealth property in blood circulation and improved stability in plasma	[84]
Diethylaminomethyl-dextran	Drug	- Improved the stability of liposomes and enhanced skin permeation	[85]
Hydroxypropyl methylcellulose	Sildenafil	- Provided greater bio-adhesion and higher entrapment efficiencies for the liposomes - Prolonged the drug release	[86]
**Polymer/Copolymer**			
Polyethylene glycol PEG	Vitexin	- Increased the stability of long drug carriers - Increased the drug entrapment efficiency and obtained a delayed release	[17,87,88]
Poly (hydroxyethyl-l-asparagine)	Antitumoral drugs	- Enhanced the stability and the bioavailability of the drug - Improved long circulation properties and the precise targeting of drug	[89,90]
Poly(L-lysine) and poly (L-glutamic acid)	Recommended for drug formulations	- Increased liposome stability in the biological environment	[91]
Eudragit EPO	Curcumin	- Prolonged the release of the drug and improved the bioavailability upon oral administration - Improved the in vivo activity and enhanced the stability in the gastrointestinal tract of the active compound	[92]
**Proteins**			
Whey protein	Astaxanthin, iron	- Improved the physical properties of liposomes: higher thermal stability, monodisperse distribution, and high encapsulation efficiency	[93,94,95]
Albumin	Vancomycin/paclitaxel/ellagic acid	- Used as a drug-binding molecule to improve the long blood retention and biocompatibility - Increased the long release of the drug, showing superior deliverability for substances, high stability, effective loading content, and high capacity of controlled targeting	[96,97,98]
Zein	Indocyanine green	- Improved the chemical stability and storage stability of liposomes	[99]
Silk fibroin (SF)	Ibuprofen	- Sustained ocular drug release and in vitro corneal permeation	[100]
Gelatine	Arginyl-glycyl-aspartic acid/aspartic acid/*Lactobacilli rhamnoses*/Amphotericin B	- Increased the efficiency of drug delivery - Obtained small-sized liposomes, physically stable, with high drug encapsulation efficiency	[101,102]
Collagen	Dexamethasone	- High potential for use as drug delivery systems for implant substances that can induce bone and cartilage differentiation	[103]
**Combinations between groups of materials**			
Chitosan–gelatine	ω-3 PUFA/BSA	- Enhanced the physical and chemical stability of liposomes - Improved the release of the active compound	[104,105]
Chitosan–sodium alginate	Inactivated PR8 Influenza virus/cationic liposomes/resveratrol	- Improved the long release of the virus and improved resistance during digestion - Improved liposome structures	[106,107,108]
Chitosan–pectin	Neohesperidin/norfloxacin	- Increased resistance during digestion of liposomes - Improved the liposomes’ physical and chemical stability	[109,110]
Chitosan–arabic gum	5I-1H-indole (5ID)	- Improved the solubility and sustained the release of the antifungal drug	[111]
Chitosan–xanthan gum	Pulmonary drugs	- Improved physical and chemical properties and storage stability of liposomes - Prolonged the release of the active compound	[112]
Chitosan–zein	*Pulicaria gnaphadoles (Vent) Boiss*	- Prolonged the antimicrobial and antioxidant activity of the active compound - Controlled release of the active compound	[113]
PEG–chitosan	Doxorubicin/Stearoyl spermine	- Increased stability and prolonged the release of the drug	[114]
Pectin–whey protein	Negatively and positively charged liposomes	- Improved the physical and chemical stability of liposomes - Protected the liposomes during gastrointestinal digestion	[115]
Pectin–polygalacturonic acid	Nisin	- Sustained the release of the active compound and improved the physical and chemical properties	[71]
Whey protein, xanthan gum, tragacanth, arabic gum, and sodium alginate	Gingerol	- Provided proper cell protection against oxidative stress - Increased the stability of the liposomes during storage - The liposomes maintained their structures in acidic and neutral media	[116]
**Other**			
Genipin (glycoside, cross-linker)	Flaxseed oil/perilla oil/tannic acid	- Increased the bioavailability and prolonged the release of the drug - Improved physical and chemical properties, long-term stability, and in vitro release of the active compound - Obtained small-sized liposomes with high encapsulation efficiency and improved biocompatibility	[51,72,117,118]
Silica	Epirubicin-hydrochloride/Alfa-choriogonadotropin	- Used for the potential oxygen protection of liposomes - Enhanced the stability of drug-loaded vesicles in the gastrointestinal tract and the photovoltaic stability	[119,120]
Calciumcarbonate	Drug	- Increased stability and reduced leakage due to the continuity of smooth and uniformly coated liposomes	[121]
Ceramic	Cholesterol	- Good stability and anti-interference ability with a good practical application for liposomes	[122]

**Table 4:** Coating materials for liposomes with applications in the food industry.

Coating Material	Type of Encapsulated Food-Grade Active Compounds	The Advantage of the Coating Material Used	Bibliography
**Saccharides and their derivatives**			
Chitosan	Fish-derived peptide/glutathione/caffeic acid/flavonoids/quercetin/sour cherry extract/*Morus nigra* waste extract/caffeine	- Enhanced the thermal stability and maintained the antioxidant activity of the peptide fractions - Protected the synergistic antioxidant effect of the active compounds - Improved stability and the antioxidant and anti-proliferative activities with reduced syneresis were protected - Improved bio-accessibility and sustained the release of active compounds in the digestive system	[126,127,128,129,130]
Sodium alginate	Vitamin C	- Increased the microbiological stability of liposomes - Enhanced the controlled release property	[83]
Calcium alginate	Enzymes	- Improved the functional properties of the liposome carriers due to increased surface area	[155]
Pectin	Echinacosides and verbascoside	- Provided an improved release in vitro digestion	[156]
Inulin	Without active compound	- Showed improved thermal, physical, and oxidative stability of liposomes	[157]
Lactose	Quercetin	- Improved conservation and release of antioxidants	[154]
**Proteins**			
Whey protein	Astaxanthin	- Improved thermal and light stability of liposomes - Improved the bio-accessibility of the active compounds and the stability under gastric conditions	[68,69]
**Combinations between groups of materials**			
Chitosan–sodium alginate	Polyelectrolyte	- Improved physical and in vitro digestion stability of the liposomes	[83]
Chitosan–pectin	*Hibiscus* extract	- Improved physical and in vitro digestion stability of the liposomes	[158]
Chitosan–zein	*Pulicaria gnaphalodes (Vent)* extract/quercetagetin	- Prolonged antimicrobial and antioxidant activity of the encapsulated compound - Improved light, thermal, and storage stability of liposomes - Enhanced the controlled release of active compounds in digestion	[113,159]
Pectin–whey protein	Antimicrobial peptides	- Protected the liposomes against gastrointestinal digestion	[90]
**Others**			
Poly (L-lysine)	Bacteriophage/bioactive peptides	- Improved chemical and thermal stability of liposomes - Improved antimicrobial properties of the active compound	[160]

**Table 5:** Recent clinical trials with coated liposomes.

Drug	Formulation/Coating Technology	Used for	Expected Results after the Clinical Trial	Phase	Clinicaltrials.gov Identifier
Bupivacaine	Liposomal bupivacaine/DepoFoam technology	Opioid use pain, postoperative colectomy colorectal surgery	A substantially longer duration of action than normal bupivacaine (96 h versus 8–9 h, respectively)	IV	NCT03638635
2B3-101- (glutathione (GSH) pegylated liposomal doxorubicin hydrochloride formulation)	PEG coating	Meningeal carcinomatosis	Coating liposomes with PEG guarantees that they circulate for a longer period of time in plasma	II	NCT01818713
Anti-EGFR immunoliposomes loaded with doxorubicin	Attached are monoclonal antibodies or antibody fragments to the surface of liposomes (immunoliposomes, antibody-linked nanoparticles)	Solid tumors	Immunoliposomes coated with antibodies bind more selectively to antigens expressed on target cells, and they are internalized more efficiently. Drug resistance can be overcome by such delivery systems	I	NCT01702129
Liposomal astaxanthin	Not specified	Bioavailability of astaxanthin formulations	Human crossover pharmacokinetic study pathways of astaxanthin in the bloodstream	Not applicable	NCT02397811
Doxil	Not specified	Breast cancer	Assess the effectiveness of using heat therapy in addition to the chemotherapy drug Doxil to treat recurrent breast cancer that has spread to the chest wall after mastectomy	II	NCT02192021

**Table 6:** Recent patent applications and patents on coated liposomes.

Patent Application/Patent, ID, Title, Year	Material Coating	Effects on Stability
Ceftazidime combined powder injection and preparation method and product specification thereof, CN111840232A, 2020	The ceftazidime and chitosan nanoparticles are each coated with vesicles, and the liposomes are then combined to create liposome-mixed nanoparticles	The product purity is high, the selection range is expanded, the side effects are minimal, and the safety is high. The product obtained by the preparation process is of stable quality and good pharmacological effect.
Treatment of age-related macular degeneration, US2020262903A1, 2020	The nanoparticles are coated with a drug targeting the vascular endothelial growth factor receptor (VEGFR), such as anti-VEGFR antibodies, anti-VEGFR aptamers, anti-VEGFR binding peptides	The stability of the nanocomposite at elevated temperatures indicates the successful support of GOF for liposomes
A biodegradable nano-theranostic composite and process of preparation thereof, US2020237667A1, 2020	Graphene oxide (GO) was deposited in the form of a thin film on both the inner and outer surfaces of the liposomes	The stability problem was solved by reinforcing the very fragile lipid membrane-based liposome wall with a dense inclusion of GO. This makes the wall very stable, even at a pH as low as 5 for several hours and at temperature as high as 50 ᵒC
DNA brick-assisted liposome sorting US20210267894A1, 2021	Liposomes were coated with DNA	The stability of a liposome was improved and more functionalization was possible when a DNA coating was applied. DNA coatings have been useful because nucleases can easily remove them, and they are inert to most biochemical reagents.
Liposomes encapsulating anticancer drugs and use thereof in the treatment of malignant tumors US20050100590A1, 2005	Liposomes were coated with a lipopeptide consisting of a lipid fragment, an active oligopeptide, and an oligopeptide spacer between the other two fragments	The addition of a negatively charged phospholipid favors the stability of the liposome solution and prevents the spontaneous aggregation of the liposomes
Liposome-based mucus-penetrating particles for mucosal delivery US20170281541A1, 2017	Liposomes were coated with PEG	PEG was used to increase the stability and solubility of liposomes with drugs, reduce toxicity, and prolong the half-life
Method of producing immunoliposomes and compositions thereof US20090232730A1, 2009	Liposomes were coated with hyaluronan/hyaluronic acid or other glycosaminoglycans	Hyaluronan/hyaluronic acid provided protection against lyophilization and reconstitution so that only nanoscale liposomes covalently coated with hyaluronan/hyaluronic acid were structurally preserved
Liposomal formulations for delivery of nucleic acids US10583084, 2020	Liposomes were coated with a glycosaminoglycan (hyaluronic acid)	The coating materials improved the condensation and stability of the liposomes

**Table 7:** Liposome commercial products available on the market.

Class of Active Compounds	Active Compounds Encapsulated	Commercial Name(Company/Country)	Route of Administration
**Pharmaceutics**			
Protease inhibitor	Amprenavir	Agenerase^®^ (GlaxoSmithKline/United Kingdom)	Oral product
Protease inhibitor	Ritonavir	Norvir (Abbott laboratories/United States of America)	Oral product
Protease inhibitor	Saquinavir	Fortovase^®^ (Hoffmann-La Roche lnc/Switzerland)	Oral product
Antitumor antibiotic	Doxorubicin	Doxil (Sequus Pharmaceuticals, Inc.,/United States of America), Evacet, Lipo-Dox, DC99^®^ (Liposome Company NJ/United States of America)	Injectable product
Antitumor antibiotic	Daunorubicin	DaounoXome™ (NeXstar Pharmceuticals, Inc., Co/United States of America)	Injectable product
Antitumor antibiotic	Irinotecan	Onivyde^TM^ (PharmaEngine/Taiwan)	Injectable product
Anti-inflammatory drug	Ibuprofen	Ibunex (Phoenix Pharma Pvt. Ltd./India), Solufen^®^ (Sanofi- Aventis/France)	Oral product
Antihypertensive	Atorvastatin	Lipirex^®^ (Sanofi-Aventis/France)	Oral product
Immunosuppressive	Cyclosporine	Neoral^®^ (Novartis/Switzerland)	Injectable product
Local anesthetic	Lidocaine	ELA-Max (Ferndale Pharmaceuticals Ltd./United States of America)	Oral product
Antifungal	Amphotericin B	Abhope (Abbott laboratories/United States of America), Ambilon (Celon Pharma Ltd./Poland), Abelcet (Liposome Company NJ/United States of America), AmBisome (Astellas Pharma Inc.,/United States of America, NeXstar Pharmceuticals, Inc., Co/United States of America), Amphocil (Sequus Pharmaceuticals, Inc.,/United States of America), Myocet^®^ (GP-Pharm/Spain), Amphonex (Bharat serums & vaccines ltd/India)	Injectable product
Photosensitizing agents	Verteporfin	Visudyne^®^ (Bausch & Lomb Incorporated/United States of America)	Injectable product
Antimetabolite antineoplastic agent	Cytarabine	DepoCyt^®^ (Pacira Pharmaceuticals, Inc/United States of America)	Injectable product
Chemotherapy drug	Cisplatin	Lipoplatin^®^ (Regulon, Inc/Greece)	Injectable product
Opioid	Morphine sulfate	DepoDur^®^ (Skyepharma Production SAS/France)	Injectable product
Antibiotic	Amikacin	MiKasome^®^ (NeXstar Pharmceuticals, Inc., Co/United States of America)	Injectable product
**Dietary/Food supplements and nutraceuticals**			
Vitamins and minerals	Vitamin C	Liposomal Vitamin C (Hypernatura^®^/Romania, WeightWorld/United Kingdom, NutriFlair/United States of America, Curesupport/Netherlands), Altrient C (LivOn Labs/United States of America), Vitamin C Liposomal (Actinovo Actinovo/Germany, Laboratoire Biocyte/France)	Oral products
	Vitamin D3	Liposomal Vitamin D3 (Lipolife/United Kingdom, California Gold Nutrition/United States of America, Dr Mercola/United States of America), Mega-Liposomal Vitamin D3 (Aurora Nutrascience/Canada), Vitamin D3 Liposomal (Laboratoire Biocyte/France)	
	Zinc	UltraZin^®^liposomal zinc (Laboratoire Biocyte/France), Liposomal zinc (AbelaPharm/Serbia)	
	Minerals	Cal/Mag/Zinc Liposomal (Laboratoire Biocyte/France)	
	Multivitamins and minerals	Lipozomal Vegan D3 K2 Magneziu (Hypernatura/Romania), Liposomal multivitamin (GymBeam/Germany), VENTUS liposomal Omega 3, Omega 6 (Life Spirit/United States of America), LVC5 (Lipolife/United Kingdom)	
Proteins	Collagen	Collagen liposomal (Actinovo/Germany, Healthydrops/North America), Collagen Zooki (YourZooki/United States of America)	
	Glutathione	Liposomal Glutathione (Hypernatura, Lipolife), Glutathione Liposomal (ActiNovo/Germany, Laboratoire Biocyte/France), Altrient Glutathione (LivOn Labs/United States of America)	
Polyphenols	Resveratrol	Liposomal Resveratrol (CureSupport, Lipolife/United Kingdom, Actinovo/Germany, Healthydrops/North America,), LLR1 Liposomal Resveratrol (Lipolife/United Kingdom)	
	Quercetin	Quercetin Liposomal (Actinovo/Germany), Liposomal Bio Quercetin (DesBio PAO/Germany)	
	Antioxidant mix	Liposomal NMN (Codeage/United States of America), HistX (Lipolife/United Kingdom)	
	Curcumin	Micelle Liposomal Turmeric (Purality Health^®^/United States of America), Curcumin Lipozomal (Actinovo/Germany, Somavita^®^/United States of America), Liposomal curcumin (Hypernatura^®^/Romania, Lipolife/United Kingdom, Healthy Drops/United States of America), Liposomal Curcumin C3 LIPOSOL (SABINSA/United States of America)	
Others	γ-Aminobutyric acid, melatonin, magnesium	Lipozomal Sleep Formula (Hypernatura^®^/Romania)	
	MSM, glucosamine, Boswellia	Lipozomal Joint Formula (Hypernatura^®^/Romania)	
**Food products**			
Functional teas	Plant sterols, EGCG, choline, potassium	Cholesterol Aid (Bio-Up Mimetic Technologies Codeage/United States of America)	Oral product
	Omega-3, CoQ10, choline, EGCG	Cardio Vitality (Bio-Up Mimetic Technologies Codeage/United States of America)	

## Data Availability

No new data were created or analyzed in this study. Data sharing is not applicable to this article.
